# Failure to define low back pain as a disease or an episode renders research on causality unsuitable: results of a systematic review

**DOI:** 10.1186/s12998-017-0172-9

**Published:** 2018-01-09

**Authors:** Emad M. Ardakani, Charlotte Leboeuf-Yde, Bruce F. Walker

**Affiliations:** 1 School of Health Professions, Murdoch University, 90 South St, Murdoch, WA 6150 Australia; 2 Institute for Regional Health Research, University of Southern Denmark, Odense, Denmark

**Keywords:** Low back pain, Cause, Risk factor, Onset, Incidence, Systematic review, Methodology

## Abstract

**Background:**

Causative factors may be different for the very first onset of symptoms of the ‘disease’ of low back pain (LBP) than for ensuing episodes that occur after a pain-free period. This differentiation hinges on a life-time absence of low back pain at first onset and short-term absence for further episodes. In this systematic review, we explored whether researchers make these distinctions when investigating the causality of LBP.

**Methods:**

A literature search of PUBMED, CINAHL, and SCOPUS databases was performed from January 2010 until September 2016 using the search terms ‘low back pain’ or ‘back pain’ and ‘risk factor’ or ‘caus*’ or ‘predict*’ or ‘onset’ or ‘first-time’ or ‘inception’ or ‘incidence’. Two reviewers extracted information on study design, types of episodes of back pain to distinguish the disease of LBP and recurring episodes, and also to determine the definitions of disease- or pain-free periods.

**Results:**

Thirty-three articles purporting to study causes of LBP were included. Upon scrutiny, 31 of the 33 articles were unclear as to what type of causality they were studying, that of the ‘disease’ or the episode, or a mere association with LBP. Only 9 studies used a prospective study design. Five studies appeared to investigate the onset of the disease of LBP, however, only one study truly captured the first incidence of LBP, which was the result of sports injury. Six appeared to study episodes but only one clearly related to the concept of episodes. Therefore, among those 11 studies, nine included both first-time LBP and episodes of LBP. Consequently, 22 studies related to the prevalence of LBP, as they probably included a mixture of first-time, recurring and ongoing episodes without distinction.

**Conclusion:**

Recent literature concerning the causality of LBP does not differentiate between the ‘disease’ of LBP and its recurring episodes mainly due to a lack of a clear definition of absence of LBP at baseline. Therefore, current research is not capable of providing a valid answer on this topic.

## Introduction

### LBP in perspective

Low back pain (LBP) is a common musculoskeletal condition in the general population and one of the five most common causes of disability worldwide [1]. It accounts for a considerable amount of healthcare visits and treatments, which places a great burden on health budgets in many countries [2]. Hence, it has been a condition of attention among various professions, consumers, and policy makers in the healthcare sector. It is believed that most cases of LBP are non-specific [3, 4], in which no clear structural or anatomical cause can be identified.

Further, those with LBP at one point in time will have a strong tendency towards having continuing LBP or having it again, and those without LBP are unlikely to develop it [5].In fact, it has been shown to be a chronic disease [6–9], characterised by a stable pattern of episodes [5], which may occur frequently or rarely [10] and these episodes may be of short or of long duration [11, 12].

A search of the Cochrane library using the search term “low back pain” reveals many systematic reviews that conclude that no specific treatment has been found to be significantly superior in the management of non-specific LBP than others, or indeed, even better than placebo [13–20], indicating a need to initiate a shift in the research direction from treatment to prevention.

### Concepts of risk factors, cause, and prevention

A risk factor, as used in epidemiologic studies, is defined as “a factor that is causally related to a change in the risk of a relevant health process, outcome, or condition. The causal nature of the relationship is established on the basis of scientific evidence and causal inference.” [21] This means that risk factors should be present prior to the onset of the disease in order to be considered causative. However the fact that one factor precedes another does not necessarily imply causality [22]. Causative factors must be avoidable or, at least, modifiable to make prevention possible.

If a suspected risk factor is found to occur concomitantly with a disease, it infers association and not causation, unless it is certain that the suspected risk factor was present before the disease onset. Therefore, in the case of mere associations, it is not known if the suspected risk factor is actually causal or only a so-called risk marker or risk indicator [21]. Hence, usually a prospective study design is required to be able to ascribe a ‘cause’ to a disease. Other tenets, in addition to associations and temporality, are needed to establish with some certainty the causal link between two variables, as so eloquently described by Bradford Hill [22].

### Prevention of chronic recurring diseases

In the case of a chronic recurring illness, prevention can be aimed at either the onset of the disease or the onset of episodes. In back pain, there seems to be some confusion on this issue. Back pain is described by many not as a ‘disease’ but a ‘symptom’, and it is accurate to say that ‘symptoms’ are the manifestations of a ‘disease’, so these two concepts should be separated. This is of great importance because causative factors are possibly quite different for the disease itself than for its various episodes, when symptoms become apparent. Therefore, it is also reasonable to postulate that the preventive approach may be different for the disease than the episodes of symptoms.

### Comparison of LBP with asthma and migraine

Asthma and migraine are both chronic recurrent diseases, characterised by episodes and often a total absence of symptoms in between. Both are likely to have a different set of causes for the *disease* of asthma and the *disease* of migraine as opposed to the subsequent symptoms (*episodes*). Thus, there would be an intrinsic reason why asthma and migraine are present, whereas external factors often trigger the onset of the symptoms/episodes.

It is possible that for back pain – also a chronic, recurrent disorder - the situation is the same. Therefore, some people have the disease of back pain, just like others have the disease of asthma. For those having an underlying predisposition to episodes of back pain or asthma attacks, it would be possible to identify factors that bring on these symptoms or episodes. In back pain, this could be awkward work positions, inactivity or sudden movements, whereas in asthma it could be exertion or exposure to smoke or cold air. If these triggering factors can be avoided, then it would be possible to reduce the number of episodes. Although episodes of both asthma and migraine can mostly be successfully treated and often also prevented, there is, as yet, no preventive approach known to be able to prevent these ‘diseases’, because the underlying predispositions for these asthmatic and migraine episodes are unknown. Therefore, it is critical that research on causes of back pain, asthma, and migraine determines clearly whether to target the onset of the ‘disease’ (i.e. the underlying predisposition) or the symptoms (i.e. the episodes).

### Importance of the disease-free and episode-free periods

To search for disease causation, truly ‘disease-free’ people should be followed over time to identify those who develop the disease, and the potential risk factors must be shown to be present before the disease commences. Recall bias and memory decay suggest it is not sufficient to ask study participants if they had pain in the last month or the last year and, if the answer is ‘no’, we cannot assume, as some authors appear to, that they never had the disease prior to that time. The study of the causes of the ‘disease’ of LBP requires access to people completely free of it, as this prevents contamination by those who were merely in a pain-free period between episodes of back pain. This is obviously not an easy task.

Similarly, an investigation into the causes of episodes requires that ‘episode-free’ people be studied over time. This necessitates a clear definition of both ‘pain-free’ and the length of this pain-free period.

Therefore, the definition of what constitutes the primary health problem in question and what can be considered to be an episode-free period prior to the start of the study must be clearly understood. That is, life-long absence of symptoms, in the case of the study of the ‘disease’, and absence of symptoms in between episodes, in the case of the study of the episodes. Otherwise, a mixture of these two concepts results in ambiguity, as it will mix causative factors for the ‘disease’ with the causative factors for its episodes. In conclusion, it is important to separate the search for causes of the ‘disease’ from the search for the causes of episodes.

### Causal research disregards the concept of disease vs. episode

It is often stated in the literature that causes of LBP are largely unknown, which probably means that the causes of the disease of LBP are unknown. Nevertheless, concerning LBP research, the search for causative factors seems to disregard the concept of the ‘disease’ vs. episode. A multitude of studies have been conducted over past decades on the topics of ‘cause’, ‘risk’, and ‘prevention’, seemingly without a clear distinction of whether these studies related to the disease of LBP, its episodes, an undefined mixture of both, or perhaps even constant and long-lasting LBP without any remissions.

### Prospective design is not the same as temporality

Some of these LBP studies seem to be carried out based on the concept that a prospective design guarantees that the statistical association between two variables is causative, disregarding the rule of prior absence of disease. Also some authors seem to ignore the concept of temporality (a fundamental causation tenet previously described by Bradford Hill [22]), but use the term ‘risk factor’ for variables that were found to be present at the same time as the disease under investigation. This is at odds with the principle of a causal relationship, as these factors can only be interpreted as a ‘risk marker’ or a potential ‘risk indicator’. In other words, they could possibly be true causative agents, but the study design can only attribute their presence as a statistical association.

### Aims and objectives

To alert the research community to these two concepts (disease vs. episode and risk factor vs. risk marker or indicator), we performed a systematic review of contemporary studies that purported to investigate the causes of LBP. Our overall aim was to see if a sample of recent studies on the causes of LBP differentiate between disease and episodes and if their approach makes it possible to draw a conclusion on causality for these specific outcomes. Our specific objectives were to:

1. Establish the proportion of included studies that used a prospective study design, which would usually be necessary in order to establish causality.
2. Determine whether included studies were concerned with the onset of the disease of LBP or the onset of episodes, or if they failed to identify the appropriate target group in this respect.

## Methods

### Search strategy, inclusion criteria, and exclusion criteria

The PRISMA statement was used to assist the methods for the study. A literature search of PUBMED, CINAHL, and SCOPUS databases was performed for contemporary studies. We arbitrarily selected the period from January 2010 until September 2016. Articles containing the following keywords were included: (low back pain OR back pain) AND (risk factor OR caus* OR predict* OR onset OR first-time OR inception OR incidence). An additional citation search was performed on retrieved articles’ reference lists.

The purpose of the literature search was to identify recent studies that investigated the potential causes of nonspecific LBP. The cause was considered the topic of the articles when words such as: risk factor, cause, predict were used in the title, abstract, or in the study objectives.

We did not intend to capture all existing literature on this topic; we were only interested in obtaining a fairly representative group of articles from the contemporary literature, in order to gain an understanding of the way researchers, in general, approached the topic under scrutiny. We included only full-text articles written in English.

Our exclusion criteria were: case reports, systematic reviews, study populations with specific LBP (where the pain can be assigned to a known specific pathology such as a disc herniation, spinal stenosis, infection, fracture, tumour, etc.), LBP with non-organic signs and symptoms, study populations seeking secondary/tertiary care, LBP studied in a special population (e.g. people with autism, Parkinson’s disease or pregnant women), and studies investigating only chronic/persistent LBP. Regarding the exclusion of exclusive studies of chronic/persistent LBP, we believe it is possible that risk factors are different for this type of LBP. Importantly, we were interested to understand if researchers had implemented a clear definition of an episode of LBP (either the very first or a recurrent one). Therefore, the presence of a pain-free period was crucial in this regard. This phenomenon will not frequently happen in patients with chronic persistence LBP.

**Fig. 1:**
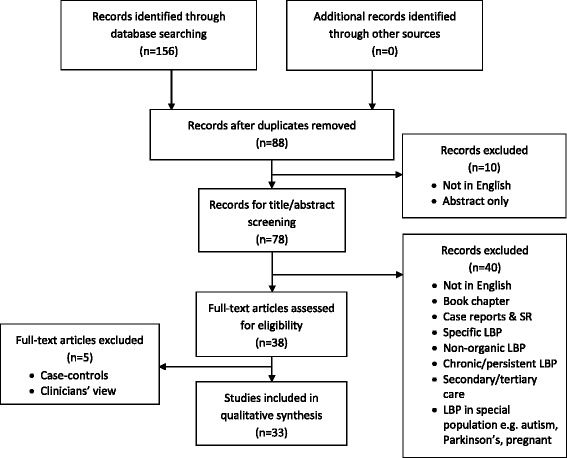
Flow chart of search results

Throughout the selection process, it was decided to exclude case-control studies because of their retrospective approach [23–26] and those studies in which clinicians’ views were sought [27] (Fig. 1).

### Article inclusion and selection process

One reviewer (EA) conducted the database search and removed all duplicates. Two reviewers (EA and CLY) independently screened titles and abstracts to identify potentially eligible studies based on the selection criteria. Articles selected by either reviewer were included for the full-text assessment. The full-texts of all selected articles were then screened independently by the two reviewers for inclusion in the data extraction phase. Articles excluded by both reviewers were excluded from the review. Where any disagreement between the two reviewers occurred and could not be resolved via a discussion and consensus approach, a third person/author (BW), not involved in the selection process, would be asked to adjudicate.

### Data extraction

A data extraction form (checklist) was developed for extracting relevant characteristics of included studies (Table 1). The rationale for these items is described below. The form was piloted by the reviewers using six articles, and some modifications were made to better suit the aims and objectives of the review. Both reviewers independently participated in data extraction and disagreements were resolved via discussion between the two reviewers. In the case of continued disagreement, the third author could be consulted.

**Table 1:** Characteristics of included Studies on causes of LBP

First author, Year, Country	Study design	Anticipated finding(s) based on the study aim(s)	Recall period for LBP Definition^a^	According to authors or obvious from text: 1st time episode, Episode, or Prevalence ^b^	Definition of preceding non-episode^c^	Risk factor data collected prior to the onset of LBP variable
Aggarwal 2013 India [28]	Cross-sectional	Prevalence, associated risk factors	1 year	Prevalence	None	No
Alperovitch 2010 Israel [29]	Cross-sectional	Prevalence, associated risk factors	1 year	Prevalence	None	No
Auvinen 2010 Finland [30]	Longitudinal	Risk factors	6 month	Episode	No LBP previous 6 month at 16 & 18	Yes for the 2nd survey
Capkin 2015 Turkey [31]	Cross-sectional	Prevalence, risk factors	Lifetime, 1 year	Prevalence	NA	No
Cho 2012 South Korea [32]	Cross-sectional	Prevalence, risk factors	Lifetime, Point, 6 month	Prevalence	NA	Not clear
Coenen 2013 the Netherlands [33]	Prospective cohort	Associated risk factor	1 year	Prevalence	NA	Not clear
Erick 2014 Botswana [34]	Cross-sectional	Prevalence, risk factors	1 year	Prevalence	NA	No
Ernat 2012 North America [35]	Retrospective, Database analysis	Incidence, risk factors	NA	Initial visit for lumbago	NA	No
Gaowgzeh 2015 Saudi Arabia [36]	Cross-sectional	Prevalence, risk factors	Undefined	Prevalence	None	No
Jia 2016 China [37]	Cross-sectional	Prevalence, risk factors	1 year	Prevalence	NA	No
Kanchanomai 2015 Thailand [38]	Prospective	Annual incidence, risk factors	3 month	Incidence	None	Not clear
Katsavouni 2014 Greece [39]	Cross-sectional	Risk factors	1 year	Prevalence	NA	No
Knox 2014 North America [40]	Retrospective Database analysis	Incidence, risk factors	NA	First diagnosis of LBP	NA	No
Lin 2014 Taiwan [41]	Cross-sectional	Prevalence, risk factors	1 year	Prevalence	NA	No
Lin 2012 Taiwan [42]	Cross-sectional	Prevalence, risk factors	Lifetime, point	Prevalence	NA	No
Mikkonen 2016 Finland [43]	Prospective cohort	Risk factors	6 month	Episode	No LBP past 6 month at 16 & 18	Yes for the 2nd survey
Mitchell 2010 Australia [44]	Prospective	Risk factors predicting new-onset LBP	Life-time, 1 year	Episode	No significant LBP at baseline	Yes
Mohseni-bandpei 2011 Iran [45]	Cross-sectional	Prevalence, risk factor	Point, 1 month, 6 month, year, lifetime	Prevalence	NA	No
Murtezani 2011 Kosovo [46]	Cross-sectional	Prevalence, risk factors	1 year	Prevalence	NA	No
Ng 2014 Australia [47]	Cross-sectional	Prevalence, risk factors	Lifetime, point	Prevalence	NA	No
Nissen 2014 Denmark [48]	Retrospective	Associated Risk factors	1 year	Prevalence	NA	No
Noda 2015 Sri Lanka [49]	Cross-sectional	Prevalence, risk factors	4 weeks	Prevalence	NA	No
Ramond-Roquin 2015 France [50]	Prospective cohort	Risk factors	1 week	Prevalence, episode	NA	Yes for the 2nd survey
Shemory 2016 North America [51]	Retrospective Database analysis	Risk factors	NA	Incidence	NA	No
Sikiru 2010 Nigeria [52]	Cross-sectional	Prevalence, risk factors	1 year	Prevalence	NA	No
Steffens 2015 Australia [53]	Case-crossover	Risk factors	NA	Episode	Free of pain in the past month	No/purposely retrospective
Sterud 2013 Norway [54]	Prospective	Risk factors	4 weeks	Prevalence	NA	No
Triki 2015 Tunisia [55]	Retrospective	Prevalence	No LBP before current injury	First LBP injury	NA	No
van Hilst 2015 the Netherlands [56]	Cross-sectional	Prevalence, associated risk factors	1 year	Prevalence	NA	No
Vandergrift 2012 North America [57]	Prospective	Risk factors	1 year	Episode	Pain-free at baseline	Yes for the follow-up survey
Vargas-Prada 2013 Spain [58]	Prospective	Incidence, risk factors	1 year	Episode	Free of pain in the past month	Yes for the follow-up survey
Vilar Furtado 2014 Brazil [59]	Cross-sectional	Risk factors	3 month	Prevalence	NA	No
Yue 2012 China [60]	Cross-sectional	Prevalence, risk factors	1 year	Prevalence	NA	No

^a^The period in which an episode of LBP was investigated for its risk factors

^b^*1st time episode*: when the very first episode of LBP was investigated, *Episode*: when any pain-free period preceding the recurrent episode under scrutiny could be identified, and *Prevalence*: when explicitly stated by authors or based on their objectives and method a mixture of all types of LBP was investigated

^c^An application of a pain-free period preceding the episode under scrutiny

### Rationale for choice of data extraction items

Initially, the information mentioned in the title, objectives, and methods was taken into account. Information from the result section was taken into consideration only when there was uncertainty surrounding the methodology.

Because studying cause(s) and risk factor(s) of a disease mandates a certain type of study design, we extracted information on the individual study designs. Another important item considered was the various types of an episode of back pain and whether it was a first episode ever or a recurring one. Also, we looked for a definition of a non-episode which, in turn, would help us differentiate types of episodes. Finally, we were also interested if data collection regarding risk factors had been done prior to the onset of LBP. For descriptive purposes, the recall period for LBP episodes was also recorded.

## Results

### Description of studies

As shown in Fig. 1, of the initial 156 potential articles, 33 articles were included in this review [28–60]. A description of these articles is found in Table 1 and summarised below. Of the studies reported in these articles, 12 were conducted in Asia/Middle East, 13 related to European countries/North America, 3 were from respectively Australia and Africa, and 2 from South America.

The terms that alerted us to their suitability of inclusion in our study were: “risk factor(s)” (*N* = 29) [28–40, 42, 45–52, 54–60], “risk” (N = 2) [41, 43], “predict” [44], “incidence” (*N* = 3) [35, 38, 58], “onset” (N = 2) [38, 44]. Also, one study investigated “modifiable personal factors” [44], and another study reported “triggers” of an acute episode of LBP [53].

The majority of included studies utilised a cross-sectional study design (*N* = 18, 54%), whereas the number of studies using a prospective design was 9 (27%). The other designs were found to be retrospective (*N* = 5) and case-crossover (N = 1). Although some authors [35, 40, 51] described their studies as retrospective, the process of data collection did not clearly indicate that.

To be able to scrutinise the approach researchers adopted towards the definition of the disease of LBP and its causes and risk factors, we tried to classify our included studies into three groups based on their objectives and methodology as follows:

1. Studies on the onset of the ‘disease’ of low back pain (first-time incident)

Five studies seemed to deal with the onset of the disease of LBP (i.e. its first occurrence or first episode ever) [35, 38, 40, 51, 55]. However, only three studies did attempt to capture subjects who had never had LBP before. Nevertheless, in two of these studies, the authors did not report on LBP per se but on the first-time diagnosis recorded in health care databases [35, 40]. Clearly, the first diagnosis of LBP does not necessarily signify the first episode ever of the pain. In the third one, the first-time diagnosis of LBP injuries due to sporting activity was reported [55]. In this study participants with a history of LBP were excluded, however given the LBP variable was investigated as a result of a sports injury, the study would not necessarily capture all cases of LBP.

In a prospective study of incidence and onset of LBP, participants who had been free of pain for the past 3 months were included [38]. However, the preceding pain status and course of LBP were not taken into account.

The fifth study that purported to study the onset of LBP used data from a health care database that reported the incidence of LBP based on medical records [51]. Thus their patient cohort consisted of those who were diagnosed with LBP, which means we do not know whether this was the first episode ever of back pain.

2. Studies on the onset of new episodes of low back pain (recurring LBP)

Six studies (18%) appeared to deal with risk factors for the onset of LBP episodes [30, 43, 44, 53, 57, 58].

Two of these studies defined the previous total absence of LBP as being free of pain in the past month [53, 58]. The others, either did not identify the previous pain-free period [30, 43] or focused on being pain-free at baseline [44, 57].

The first study [53] employed a case-crossover study design to investigate triggers of a new episode of acute LBP. An acute episode of LBP was defined as a new-onset of LBP of moderate intensity after at least one pain-free month.

The second study explored new episodes of LBP during the past month at both baseline and at a 12-month follow-up [58]. They defined the pain-free period to consist of 1 month prior to baseline, hence, making it possible to identify a new episode. The authors, however, give the impression that they were collecting data on incidence, i.e. information that also could include first-time ever episodes (i.e. the disease of LBP).

The third and fourth reports [30, 43], using data from one single longitudinal cohort study, described two surveys on the participants aged 16 (first study) and 18 (follow-up study), studying risk factors for LBP during the past 6 months. Both studies reported the 6-month prevalence of LBP at both ages. Their definition of ‘new LBP’ at 18 was the absence of LBP for the 6 months before the first survey. Because adolescence is the time in life when the first-time episode of LBP commonly occurs [61], i.e. the very onset of the ‘disease’, many of the reported LBP cases would fit in this category. However, a subgroup would already have experienced one or several previous episodes and could, therefore, be classified as having a subsequent episode (had there been a clear definition of prior absence of pain) when surveyed at the age of 18.

In the two prospective studies, in which participants without pain at baseline were included [44, 57], neither took a previous history of LBP into consideration. Therefore, it is not clear whether the pain appeared for the first time or was part of the course of the previous LBP.

3. Studies without a clear description of the type of LBP definition (possible mixture of first-time, recurring, and ongoing LBP)

In 22 studies, none of these two previously described possibilities (i.e. onset of either disease or an episode) were identified; hence they did, in fact, study the prevalence and not the incidence of LBP, whether ongoing or recurring. Nonetheless, some of the study participants, particularly if they were young, might have experienced their first episode ever. The recall periods for those prevalence studies ranged from 1 week to 1 year. With longer recall periods it is more likely that the LBP group would consist of a mixture of LBP definitions (i.e. first-time, recurring, or ongoing).

### Reclassification of study type based on our interpretation of reports

1. **Studies on the onset of the ‘disease’ of low back pain (first-time incident)**

None of the studies appeared truly to study the ‘disease’ of LBP. Among those five studies included in the category of first-time incident (onset of ‘disease’), four articles [35, 38, 40, 51] have the propensity to include a mixture of first-time and recurrent episodes. The fifth study, did report first-time diagnosis of LBP injuries but due to sporting activity [55]. Although participants with a history of LBP were excluded, this study would therefore not necessarily capture all cases of LBP.

2. **Studies on the onset of new episodes of low back pain (recurring LBP)**

In addition, in 5 out of 6 studies [30, 43, 44, 57, 58] in the category of new episodes, the same ambiguity arose due to the unclear definition of LBP in relation to its previous pain-free period. Only one study provided a genuinely clear definition of an acute episode of LBP and was therefore able to provide causal information for the episodic LBP [53].

3. **Studies without a clear definition of type of LBP definition (possible mixture of first-time, recurring, and ongoing LBP)**

At first observation, 22 studies did not describe adequately their LBP definition and requirements. Nevertheless, a more thorough scrutiny of the articles initially placed in the first two categories had revealed that 4 of the first 5 and 5 of the second 6, in fact, belonged to this third category. Thus, in all, 31 of our reviewed 33 studies were not able to study or differentiate the causes of the ‘disease’ or causes of episodes of LBP. Nevertheless, three studies would be able to study prediction of the first-time *diagnosis* of LBP [35, 40] with the possibility of including both first-time and recurring episodes, and one would be able to correctly identify factors predicting first-time LBP *injury* [55].

## Discussion

According to this survey, many research reports relating to the causes of LBP fail to use a prospective study design and are not explicit on what aspect of LBP they are studying, i.e. the very onset (first episode) or subsequent episodes.

While some potential risk factors can be detected using a cross-sectional design, they clearly need to be present before the onset of LBP. Examples are sex and childhood socioeconomic circumstances, but for many potential risk factors a cross-sectional association merely means that there is a statistical association. A strong dose-response would be a good indication of a causal link [22], but the fundamental issue of temporality must first be established, in order to know that the link is causal. Therefore, in this review we chose to highlight the Bradford-Hill tenet of temporality because it is an absolute criterion for causality and is particularly relevant with diseases of slow development.

Because most prospective studies typically lacked a definition of the preceding ‘non-episode’, it was impossible to know if the purported causes related to the ‘disease’ or the episodes. De Vet et al. reported, in a previous publication, that researchers use different definitions of episodes/non-episodes and that in many studies there is no definition at all [62]. Her team recommended that there should be a consensus on what is meant by “episodes” and they proposed an episode of LBP should last at least 24 h and be preceded and followed by a period of at least 1 month without low back pain. The proposition was agreed in a modified Delphi study among a panel of international experts in LBP [59]. Some studies also investigated the application of the proposed definition in primary and secondary care patients [12, 60]. Nevertheless, researchers seem to ignore or have not noticed this important recommendation.

As LBP often starts early in life, and is a recurring disorder, new cases are unlikely to occur later in life but are of course possible. Therefore, if it is unclear if study subjects, who were pain-free at baseline, were merely experiencing a pain-free ‘non-episode’ or if they had never previously had back pain at all. The younger they are, the more likely that it could be a first-time experience and the older they are, the more likely that they are experiencing yet another episode. This could perhaps be dealt with, if studies reported data distinctly for different age groups, or if study samples were representative of specific age groups.

In addition, there is a third group to contend with, namely those with persistent/chronic LBP. A recent study of the general population, using frequent text-message data collection on 50-year olds during 1 year, showed that there were three almost equally large groups: those who never or almost never have LBP, those who have it episodically, and those who almost always have it [63]. The last two groups may well have different sets of risk factors and must be separated during data collection.

In sum, the contemporary literature on the causality of LBP, with very few exceptions, is unable to bring any valuable answers to the questions of causality.

### Methodological considerations

Some possible weaknesses of our review should be acknowledged. For instance, it is not certain that all relevant articles were retrieved. However, we were not looking for the answers to causes of LBP; instead, we were interested in researchers’ recent approaches to causality, making it irrelevant to locate all relatively contemporary articles on this topic.

Another potential limitation was the search time limit. We did search for articles published from 2010. However, we wanted to include contemporary literature, which could provide us with an up-to-date general view on this topic.

There was no previously published and tested checklist suitable to our purposes. Perhaps another review team could have developed a different checklist with somewhat different results. However, our questions were simple and straightforward, so we consider this unlikely.

The extraction of data was done by two reviewers independently without any interest in the outcome. Since this topic is relatively new and literature is not clear on the definitions of types of LBP, information was often difficult to identify and extract due to lack of such definitions. Despite this, there was no occasion when a third reviewer needed to settle the disagreements, which indicates, at least, that the checklist was user-friendly. It is, of course, still possible that both reviewers misinterpreted some findings in the same direction.

## Conclusions

In our study, we concentrated on a small but important detail, namely a clear statement of whether the disease of LBP or its episodes was studied. This aspect is important when establishing the temporality, i.e. the chronological order of events. The vast majority of the contemporary literature on the causality of LBP, captured in this review was unable to yield valid answers on both the LBP disease and the continued episodes of LBP.

This issue is important from a public health perspective but also for clinicians as, presently, it does not appear possible to advise patients on the causes of LBP.

Future research perspectives include the rigorous application of clear definitions of the baseline LBP status of participants and the use of standardised criteria for both absence and presence of LBP and a respect for the concept of temporality.
